# Medical and Physical Intelligence

**Published:** 1824-11

**Authors:** 


### MEDICAL AND PHYSICAL INTELLIGENCE.

### PATHOLOGY.

1. *Diagnostic between Irritation and Inflammation of the Mucous Membranes of the Bronchiæ.*—M. NAUCHE has remarked that, in the natural state, or where there only exists an excess of irritation, the matters secreted by the different mucous membranes have always a well-marked acidity: while, on the other hand, if these membranes be inflamed, alterations of their vital properties supervene; the nature of the secretion is changed, and becomes alkaline. These two states are easily recognised by a morsel of paper tinged blue by turnsol. When the matter expectorated is acid, the paper is turned red; while it assumes a deeper blue, or is even changed from red to blue, if the matter be alkaline.

M. Nauche has examined the expectoration, with respect to its acid and alkaline character, in the diseases of the respiratory organs. He believes, from this inquiry, that they may be divided into matter produced by irritation,—an increased secretion of the mucous follicles which line the membrane of the air-passages,—and into matter, or expectoration, which results from their inflammation. He has observed that the white, mucous, frothy expectoration, which is frequently brought up in large quantities by persons in a state of agony, has always an acid character, when the air-passages have not been the seat of previous disease. This character is likewise found in the white, frothy expectoration which occurs during the whole continuance of pleurisy, whether acute or chronic; and at the commencement of pneumonia, when the matter expectorated is white, or even yellow. It is often lost in the course of this disease, and re-appears towards its decline. The acid expectoration is likewise found in emphysema of the lungs and scrofulous phthisis, when the tubercles are but little developed,—in the state called crude. It is evident, in all these cases, that the mucous membranes which furnish the expectoration are only in a state of increased excitement, and that they are in no degree inflamed. M. Nauche has likewise found acid expectoration in certain advanced cases of phthisis. He believes that this depended upon these matters being the product of an increased secretion of the mucous membrane lining the excavations formed by the tubercles.

The expectorated matters, on the contrary, are always alkaline in inflammation of the mucous membrane of the bronchiæ, and in all the cases designated by the names of acute and chronic colds, or mucous phthisis *(phthisis muqueuses)*. Although this expectoration is not regarded as purulent, it is nevertheless a kind of pus peculiar to inflammation of these membranes, and analogous to the purulent serosity which is furnished by serous membranes when in a state of inflammation.

The expectoration likewise becomes alkaline in peripneumony, when the inflammation of the pulmonary tissue communicates itself to that of the mucous membrane of the bronchiæ; and this secretion is the product of the inflammation of these two tissues.

The expectoration is likewise alkaline in phthisis pulmonalis, in the second or third stage, when the tubercles become broken down. It is usual, in such cases, to find the internal membrane of the lungs deeply altered.

It frequently happens, particularly among phthisical patients, that both these kinds of expectoration are to be found in the same vessel. That which results from an augmented excitement comes up most easily, —is white, frothy, and acid; the other is brought up with difficulty, is yellow, thick, and alkaline. In this disease, when the patient has only the former kind of expectoration, his life is often in the greatest danger.

These remarks are extracted from the work which is reviewed in the foreign department of the present Number.

2. *Hydrophobia.*—Dr. CAPELLO, of Rome, in a Memoir read before the Academy dei Linæi, affirms that the hydrophobic poison, after its first transmission, loses the power of conveying the disease. This remark, already pointed out by BADER, is confirmed by repeated experiments made by Dr. Capello, both upon dogs and other animals. A lap-dog and cat were both inoculated with the saliva of a dog, who died of *inoculated* hydrophobia: they both remained free from disease; and three years afterwards the lap-dog was again inoculated, from a dog who became rabid *spontaneously:* he then took the disease, and died.

An ox was bitten by a dog attacked with rabies; he became hydrophobic, and bit many other animals: all remained free from the affection. The dog that bit the ox also bit a child, who died about four months after, with all the symptoms of hydrophobia: with the saliva of this child a dog was inoculated, but the disease was not transmitted.

Dr. Capello relates one more history in confirmation of this fact, and which is very strong, inasmuch as the animal, which was bit by another dog belonging to the same person, becoming hydrophobic on the fifty-first day, broke the chain with which he was fastened, and escaped into the street, where he bit many persons, and the dogs of two persons (who are named), and finally disappeared among the ruins of the villa of Quintelius Varus. Not one of the persons so bitten, nor even of the dogs, had the slightest symptom of hydrophobia.

Dr. Capello gives us no satisfactory account of the origin of the spontaneous hydrophobia, which he would endeavour to persuade his readers is caused by the violence of the venereal appetite in the canine species, when restrained from indulgence. He also informs us that he has not been able to verify the observations of Dr. MAROCHETTI, relative to the pustules under the tongue; and, on this point, he therefore agrees with the physicians of Wirtemburg, who, by the desire of the College of Medicine, were ordered to examine the tongues of those men and animals who were the subjects of the disease throughout that kingdom.—(*Annali Universali*, Guigno.)

3. *Case of Menstruation from the Mammæ.*—M. BUTTNER, a practitioner at Halberstadt, has recently published a case of this kind. It occurred in an hysterical woman, who experienced the ordinary symptoms of menstruation, which went off after she had evacuated five or six spoonsful of sanguineous fluid by the nipples. This process usually lasted about six days, and was succeeded by a white mucous discharge from the same parts; which however were, during the whole time, neither swelled nor painful.—(*Journal der Praktischen Heilkunde*, Junius.)

4. *Pathology of Phlegmasia Dolens.*—M. VELPEAU has related three cases of this disease, in all of which some alteration, more or less considerable, was found at the sacro-iliac symphysis of the same side on which the limb was affected; purulent effusions in the peritoneum, particularly about the genital organs; abscesses in the diseased limb; and, lastly, a mixture of pus and coagulated blood in the veins of the limb, with evident traces of inflammation of their coats, in two of the cases. M. Velpeau regards the alteration of the symphyses as the original source of the disease, which afterwards becomes extended to the limb: the veins, according to him, being only consecutively affected, whether the pus in their cavities may have been originally formed there, or introduced by absorption. The veins were incompletely obliterated.

These facts agree with the observations of M. BOUILLARD on the causes of certain kinds of dropsy; and our readers will find, by referring to our analysis of Dr. DAVIS's paper, in the last volume of the Medico-Chirurgical Transactions, that these cases are in confirmation of his views, although his opinions are not entirely the same as those of M. Velpeau.—(*Bulletin des Sciences Medicales*, Juillet.)

### THERAPEUTICS.

5. *Efficacy of Tartar-Emetic Ointment.*—Dr. TONELLI has published an account of his experience of this remedy, preceded, however, by much theoretical reasoning on its mode of action. He asserts that, in forty cases in which he employed this remedy, thirty-one were perfectly restored, four were greatly relieved, and five died. Among the diseases for which he prescribed this ointment, were fevers, pleurisies, chronic catarrhs, rheumatism, *tubercular suppurations*, acute asthma, *two cases of vomicæ*, a threatened pulmonary consumption, *another advanced to its second stage*, and an hydrothorax.

A remarkable instance is also detailed of the perfect cure of a case of insanity by this means. The patient was a woman, forty-five years of age, in whom insanity succeeded to an attack of fever: all the remedies previously adopted had failed in affording relief, when the tartar-emetic ointment was freely rubbed in, from the crown of the head to the first cervical vertebra; the head being shaved. Under this discipline, nausea was produced, and continued constantly; a large crop of pustules soon appeared; and in two months the symptoms of mental malady had entirely ceased.

The quantity of tartar emetic employed was one drachm and a half to the same quantity of lard, sometimes a drachm to three of the ointment; and the author recommends that the tartar emetic be reduced to an impalpable powder. The parts where he employs the frictions are the epigastrium, the anterior part of the thorax, and at the same time upon the back, between the internal margin of the scapula and the vertebral column.—(*Annali Universali*, Luglio.)

6. *Effects of the Croton Oil.*—Dr. Fenoglio, of Turin, has published a series of experiments on the use of the above-mentioned oil; from which he draws the following conclusions:—

1st. That the effects of this oil are to produce a sense of burning in the fauces, slight pains in the belly, and a general sense of fatigue.

2d. That it may be looked upon as an antiphlogistic of no ordinary powers; but not to be administered where there exists any inflammatory action of the digestive canal.

3d. That it does not possess any diuretic property.

4th. That, generally speaking, in the doses usually administered, it is powerfully drastic.

5th. That, in doses of a drop, it does not produce the violent effects that have been by some attributed to it.

6th. That, where more numerous evacuations have been procured, other purgatives, or glysters, have assisted its action.

7th. That, given in the form of pill, the effect upon the mouth and fauces is avoided.

8th. That it should not be given in solution, as it appears thereby to be imperfect in its action.

So far Dr. Fenoglio's experience extends. We have, immediately following, the result of experiments made in the clinical department of the University of Padua, communicated to us by Pietro Benvenuti, and which appear more in conformity with the experience of the English practitioner. The conclusions this latter gentleman has arrived at are the following:—

1st. That the Croton oil is the most violent of all drastic purgatives at present known.

2d. That this oil produces its effects without the medium of any vehicle (by mere contact).

3d. That the irritation of the fauces produced by it, is always greatest when the oil is least diluted.

4th. That half a drop operates in a much greater degree of proportionate violence than a larger quantity.

5th. That the purgative effects are in an inverse ratio to the irritation produced upon the fauces.

6th. That it is possessed not only of an acrid, but of a caustic quality also.

7th and lastly. That, besides its action on the fauces and intestines, it appears to exert a power over the urinary apparatus, *diminishing* the quantity of urine; and, therefore, the Professor thinks that it may be given with every chance of success in cases of Diabetes mellitus.—(*Annali Universali,* May.)

---

### SURGERY.

7. *New Operation, proposed as a Substitute for the Cæsarean Section.*—M. BAUDELOCQUE, nephew of the celebrated accoucheur of that name, proposes to substitute the following method, to which he gives the name of *gastroelytrotomie*, for the Cæsarean operation.

The position is the same usually adopted: the woman is to be placed on an horizontal plane during the first incision, which extends along the outer border of the rectus muscle, from the umbilicus to two inches above the pubis. "In the former of the two methods which I propose, I take care," continues M. Baudelocque, "not to involve the peritoneum in the external incision. I then pierce the membranes per vaginam, so as to remove the waters in this way. I make the legs and thighs be half bent, and pass the forefinger into the inferior angle of the wound, to remove the peritoneum carefully from the whole extent of the iliac fossa; and, when this membrane is entirely detached, an assistant, placed at the right side of the patient, raises up the peritoneum and mass of the intestines; whilst another, placed beside him, maintains the uterus in its position, by applying one hand upon the belly. I introduce the right hand into the abdominal cavity, so as to explore the iliac artery, and then ascertain if there be any arteries surrounding the vagina: if there be any, I apply a ligature at both extremities; and, before cutting them, I take care to recognise the sub-pubian ligament, in order to avoid it. The bladder being emptied by the catheter, if the patient be unable to void her urine, and the rectum by glysters. I smear the left hand with some kind of lard, and introduce it between pronation and supination, parallel to the axis of the inferior orifice, and easily bring the vagina out beyond the external wound. Seizing the bistoury, I cover it with the three first fingers of the right hand, and plunge it into the vagina by the external opening, and as far as possible below its insertion into the neck of the uterus, prolonging the incision to the extent of four and a half inches. This incision being made, the fœtus is expelled, by the contractions of the uterus, into the abdominal cavity, in the same way as happens in rupture of the uterus or vagina. It may be extracted with pincers, or a forceps half as long as that generally employed."

The second method differs from the first only with respect to the peritoneum, which is divided at the same time with the abdominal muscles. "In the left lateral obliquity, I make the external incision on the right side of the belly, and the rest of the operation is rendered more easy, from the section of the vagina being thus capable of greater extension. In the anterior obliquity, we may operate either on the right side or the left. I replace the uterus; and, when the placenta is detached, I assist its expulsion per vaginam, by pulling it gently by the cord,—not forgetting to support the perineum with the left hand, as in natural labour, and to make only one half of the circumference of the placenta present at the vulva, when it is voluminous. It is unnecessary to say, that the process which I describe would not answer if an exostosis closed the upper isthmus, or if a schirrous tumor of the neck of the uterus entirely occupied the vagina: the same would be the case in a hernia of the uterus."

M. Baudelocque does not conceal any of the inconveniences attached to the operation which he proposes, but he believes it to be much less dangerous than the one for which he would substitute it. Experience will show.—*(Journal Universel des Sciences Medicales,* Juillet.*)*

8. *Experiments on the Treatment of the Itch.*—An extensive series of experiments on the comparative advantages of the different methods of treatment proposed for the itch has been made, under the direction of Dr. MAURY, physician to the hospital of St. Louis. The points to be ascertained were, the length of time required,—the expense of the medicines,—their effects upon the skin,—and their comparative degrees of convenience with regard to the linen of the patients. The subjects of experiment were selected;—that is to say, those only were chosen, in whom the nature of the eruption was quite unequivocal, and who had not previously made use of any external application, nor internal remedy.

Twenty-one formulæ, with their results, are given: we subjoin four of those which appear to have cured the disease in the shortest period.

1. Camphorated liniment, of M. VARDY; composed of two ounces of olive or almond oil, and two drachms of camphor. Mean duration of the treatment, $13\frac{3}{10}$ days. This medicine is too expensive for habitual use at an hospital; it stains the linen; the smell is not unpleasant; it effects the cure without irritating the skin, and the itching is much relieved by the first application. It is recommended as a good remedy for private practice. The compound liniment of M. FOURNIER differs from the preceding only in the addition of two drachms of liquid ammonia, and the combination is favourably spoken of. The medium length of time required for the cure being reduced to $11\frac{4}{10}$ days.

2. Sulphur pomatum, of M. HELMERICK: sublimed sulphur, two parts; purified potass, one part; lard, eight parts. Two frictions are made in the day, using two ounces of the pomatum for each. Mean duration of the treatment, $11\frac{7}{10}$ days. The price of this is moderate; it soils the linen, from the excess of fat over the alkali; has some smell, but does not incommode the skin, and effects a speedy cure. It differs little from the "pommade sulphuro-alcaline," employed at the hospital of St. Louis.

3. Pomatum proposed by M. MELIER: subcarbonate of soda, two ounces; water, one ounce; olive oil, four ounces; flowers of sulphur, four ounces. Dissolve the subcarbonate in the water, and add the oil, so as to make a soap; then add the sulphur by little and little, carefully mixing it. Of this, two ounces are to be used for each friction, and these to be employed twice a-day. Mean period required for the cure, $13\frac{7}{10}$ days. This method presents the advantage of an oil and alkali united in such proportions as to form a soap, by which means it is prevented from staining the linen, and cures the eruption without irritating the skin. It is not without smell. It is suggested that camphor might be substituted for the sulphur, in the proportion of four drachms to the quantity above mentioned.

4. Sulphureous baths. To a common bath add four ounces of the sulphuret of potass. Mean time required for the cure, $17\frac{3}{10}$ days. This method is very gentle, effecting the cure without inconvenience, but slowly, and not suiting every patient. The bath may be rendered more active, and the cure more speedy, by adding a little sulphuric acid. It is expensive, however, and can scarcely be employed but on a great scale.

5. Sulphureous fumigations. Fumigations with sulphureous acid are employed at the hospital of St. Louis. The mean time required is $21\frac{4}{10}$ days. This method has been too much praised: it is expensive, and produces the cure but slowly. Many patients are unable to support it; it fatigues the chest when the lungs are weak. It is free from odour and uncleanliness; but these advantages do not compensate for the tediousness of the treatment. Spirituous fumigations are still less efficacious.

6. Decoction of tobacco; made by putting two ounces of tobacco in a pound of water, and bringing it to the boiling point. Two lotions were employed every day, consisting of half a wine-glassful each. Mean time required, $20\frac{2}{10}$ days. This method is expensive, and not altogether free from inconvenience, as several instances occurred of nausea and vertigo, while the odour proved harrassing to some of the patients.—(*Journal General de Medecine,* Juillet.)

---

### MIDWIFERY.

9. *Statements relative to the Lying-in Hospital at Florence.*—Dr. Bigeschi, giving an account of the establishment and internal economy of this hospital, which was opened in June 1816, informs us, that, up to the end of March 1824, five hundred women had been delivered in this hospital; four only having died during this period. The number of children born was 506, there being six instances of twins: of this number, 485 were head presentations; the males were 279, the females 227. Three hundred were delivered during the day, two hundred in the night, and twenty-one before the usual term of gestation. Of the whole number, three only presented any defect of formation; two having hare-lips and cleft palates, and one having a malformation of the fingers of both hands, similar to the mother. The heaviest infant weighed sixteen pounds* four ounces, and the smallest, born at the full period, five pounds; the majority weighed about ten pounds. Of the 506 children born, forty-nine perished: of these, thirteen were still-born; the others died of infantile diseases, within the first few days. In those born before the natural period, the greatest length of the fœtus was always found to be between the umbilicus and the top of the head.

The disease called induration of the cellular tissue attracted Dr. Bigeschi's particular attention. He observed it to be prevalent in the winter, especially if rigorous; in consequence of which, he ordered the infant to be kept in the mother's bed, as warm as possible, and from that time no case of the disease occurred.

Of 486 head presentations, three were extracted by means of the forceps; neither the children nor the mothers suffering any thing therefrom.

Of the four deaths, the first was occasioned by inflammation of the uterus. In consequence of malformation of the pelvis, the child was turned, with the intention of delivering by the feet, but the head being too large to pass, it was necessary to turn it, and it was finally extracted dead. In the second case, the child was delivered by the feet; and the mother, a woman thirty-one years of age, of a sanguine temperament, was seized with symptoms of inflammation of the kidneys the day after, and died on the seventeenth day of the disease: the left kidney, the spleen, and part of the lung of the left side, were found in a state of suppuration; the uterus being in a perfectly sound condition. It is remarkable that this woman had been affected, subsequent to a former labour, with symptoms indicative of the above affections; from which she did not recover for two months. The third death was occasioned by hemorrhage; the woman eight months advanced in pregnancy; the placenta was found adherent to the neck of the uterus. The head presented; the child was turned, and delivered by the feet. Every thing was conducted in the gentlest manner; but the child was still-born. The uterus contracted properly; the placenta was extracted immediately; but the patient, who had swooned at the conclusion of the operation, expired an hour after. The other patient died of puerperal fever, on the fifth day. There was nothing remarkable or preternatural in the previous labour. Dr. Bigeschi adds some observations, confirmatory of the virtues of the *secale cornutum.*

10. *Case of Anasarca combined with Pregnancy.*—Dr. De Felici, of Milan, relates the following very extraordinary case, the leading features of which we give, omitting all his speculative opinions.

Maria Orrigi, thirty-five years of age, mother of two children, had reason to believe herself pregnant in December 1817. She soon perceived a tumefaction of the feet, which rapidly extended to the thighs, the pudenda, loins, and abdomen, attended with great thirst. On the 2d of February following, she was seized with fever, and a pungent pain in the left hypogastric region, and numbness of the corresponding thigh.

---

* The Tuscan weight of twelve ounces.

These symptoms were combated by antiphlogistics, bleeding, and digitalis: nevertheless, the thirst continued insupportable; the œdema increased, the tumefaction of the abdomen became enormous; and the desire of making water was so violent and so painful, as in some measure to resemble the pains of labour, the urine only flowing drop by drop. The pain in the thigh and leg had increased greatly; there was great difficulty of breathing, palpitations, a dry cough, wasting of the upper extremities, frequent pulse, a sense of suffocation upon the slightest motion, and tumefaction of the hands and arms, as well as puffiness of the face. Such was the condition of the patient, when seen by Dr. De Felici, for the first time, on the 6th of March. Suspecting retention of urine, but seeing the threatening condition of the patient, the Doctor doubted whether he should perform paracentesis, or introduce the catheter. Fortunately, he chose the latter; and, to the astonishment of every one, drew off from thirteen to fourteen pints of urine: this operation, repeated twice during the day, produced the evacuation of an equal quantity. Nitre and digitalis were prescribed, *(the doses are not mentioned.)*

The next day, the symptoms were mitigated; the catheter was passed twice. In the evening, excepting the fever and thirst, (both of which were, however, diminished,) a change for the better had taken place: the pain in the side was gone, the thigh could be moved, and the œdema of the lower extremities was diminished by one half. The medicine had produced nausea, and was omitted.

On the following morning a purgative was taken, and many evacuations procured; but no urine was passed. The catheter drew off as large a quantity of water as on the 6th inst. About noon, labour took place, which was not followed by the slightest hemorrhage. The placenta was expelled on the following day; and from that hour there was no appearance of the lochia.

The catheter was used every two hours, on the 9th, 10th, 11th, and 12th. On the 13th, the swelling of the abdomen was entirely gone; that of the left thigh was diminishing; that of the right had entirely disappeared: the upper extremities continued still œdematous. In these eight days, 257 medical pounds of urine were drawn off. From the 14th to the 17th, a sensible diminution of the symptoms was perceptible. On the 18th, during a profuse fæcal evacuation, the patient made water for the first time: the catheter was now used every four hours. On the 19th, the patient was without fever. On the 31st, she made water naturally.

April 7th.—The patient rose from her bed, and was restored to health.—*(Annali Universali,* Luglio.*)*

11. *On the Method of counteracting Uterine Hemorrhage, proposed by* Dr. Gooch.—Since the publication of Dr. Gooch's paper, (12th volume of the Medico-Chirurgical Transactions,) on the Hemorrhage which sometimes follows the Expulsion of the Placenta and the Contraction of the Uterus, I have had an opportunity, (says Mr. Crowfoot,) in three cases, of witnessing the good effects of the preventive treatment which he recommends.

Mrs. G., in three previous labours, had suffered from a frightful hemorrhage, which followed the expulsion of the placenta, after an interval, varying in the different labours, of from five to twenty minutes, notwithstanding the uterus had actively contracted. This patient having been again pregnant, I determined to try strict antiphlogistic treatment for five or six weeks prior to her confinement. The consequence was, that she altogether escaped the hemorrhage.

Mrs. A., a lady of sanguine temperament, but delicate constitution, in her two first labours had rather more than the usual discharge after the removal of the placenta. In her third labour, the placenta was expelled as usual, the uterus actively contracted, and no hemorrhage followed for at least twenty minutes, when a most appalling one burst forth, and was with difficulty restrained by the most prompt and continued treatment. In her fourth pregnancy, the antiphlogistic treatment was pursued as in the former case, and with the same satisfactory result.

In another patient, Mrs. L., similar hemorrhages had occurred in two previous labours: in a third pregnancy, the antiphlogistic treatment prevented this inconvenience in the following labour.

In none of these cases did the hemorrhage immediately follow the expulsion of the placenta; an interval of from five to twenty minutes intervened; and I therefore infer, that a degree of relaxation of the uterus preceded the hemorrhage, and was as essential as the existence of the phlogistic diathesis. We find that the only remedies upon which we can depend in these cases, are such as have a tendency to produce firmer contraction of the organ,—the sudden application of cold to the abdomen, steady pressure externally on the uterus, and the introduction of the hand into its cavity. Dr. Gooch found that Le Roux's remedy could not be relied on; and the case which I detailed first, in which alarming hemorrhage took place, into an uterus distended by a seven-months' fœtus and its appendages, would indispose one to trust to the plugging the vagina as a means of restraining a bleeding into a dilatable organ.—*(Edinburgh Med. and Surg. Journal.)*

---

### CHEMISTRY.

12. *Digitaline.*—M. Royer has obtained the active principle of digitalis in a separate state, by the following process:—He digested a pound of the plant in ether, first cold, and afterwards heated under pressure: the solution thus formed was filtered and evaporated; the residue dissolved in water, and likewise filtered. The solution was then treated with hydrated oxyde of lead; the whole evaporated, and digested in ether. On being evaporated, it assumed the appearance of a brownish substance, of the consistence of paste, very bitter to the taste, and extremely deliquescent. Difficulty was experienced in crystalizing this; but, on dissolving it in alcohol, and evaporating it on a piece of glass over a lamp, a number of minute crystals were observed through a microscope. To determine the active nature of this substance, various experiments were tried. A grain was dissolved in 180 grains of water, and the solution injected into the abdomen of a rabbit: after a few minutes, the respiration and circulation became diminished, and the animal soon died, without apparent suffering; yet rabbits are easily affected with convulsions, from the operation of poisonous substances. Half a grain, in 120 grains of water, injected into the veins of a cat, produced death in a similar manner, in about fifteen minutes; and a dog was killed by a grain and a half dissolved in half an ounce of water, and thrown into the jugular vein.

In these cases the arterial blood assumed a venous appearance, and coagulated with difficulty.—*(Bib. Univer.)*

13. *The Effect of covering Vessels containing diluted Alcohol with Bladder.*—The result of keeping diluted alcohol under these circumstances is, that the water evaporates, leaving the spirit stronger than before. It is asserted by SOEMMERING, that if diluted spirits be put into an ox's bladder, and be thus suspended over a sand-bath, in a few days the whole of the water escapes, and nothing but pure alcohol remains.

This seems to be an important circumstance, to be kept in mind by gentlemen having extensive anatomical museums.—*(Giorn. de Fisica.)*

14. *Strychnia in the Upas tieuté.*—Both the upas tieuté and the upas anthiar have been subjected to experiment by MM. PELLETIER and CAVENTOU, who, after various trials, obtained from the former species a crystaline substance, which is thus described:—

"An aqueous solution was prepared, which, when filtered, was treated with pure calcined magnesia; the reddish yellow precipitate obtained, when washed and dried, was boiled in alcohol two or three times, and the solutions evaporated gave an orange-coloured crystalline substance. This substance was bitter, only slightly soluble in water, very soluble in acids, and had all the properties of strychnia, except that of producing a green colour with nitric acid, instead of a red one: but this effect was occasioned by the presence of a brown-coloured substance; for, when a solution of the whole was made in weak sulphuric acid, passed through animal charcoal, precipitated by magnesia, and then dissolved in alcohol, and crystallized by slow evaporation, it lost the property of becoming green by nitric acid, and was perfectly pure.

In this state, it consisted of crystalline prismatic needles, nearly insoluble in water, very bitter, restoring the blue of reddened litmus paper, saturating acids, and with them forming solutions, in which ammonia, tincture of galls, and the alkaline gallates and oxalates, produced precipitates, soluble in alcohol; and in all things, except that of reddening by nitric acid, exactly resembling strychnia. The red colour, by nitric acid, belongs therefore to some other substance than strychnia; and, on evaporating the water with which the magnesian precipitate was washed, a yellow substance, having this property, was obtained; and which, redissolved, filtered through animal charcoal, and re-evaporated, gave a tolerably pure solution of the substance. This substance is uncrystallizable, fixed, soluble in water and alcohol, and not precipitable by acetate of lead: it exists only in small quantities in the upas.

In consequence of the purity of the strychnia obtained from the upas, specimens were examined from other sources, and it was ascertained that, though most of them reddened by nitric acid, yet they varied in the extent of this property, and one very pure specimen scarcely exhibited the effect at all. Hence, it may be concluded that the red colour is always due to a portion of impurity accompanying the strychnia, and does not belong to the alkali.

The strychnia from the upas produced all the effects on the animal economy that are produced by strychnia otherwise obtained.

The brown substance, which produces a green colour with nitric acid, was found to be same as that existing in the false angustura bark; when pure, it is without taste, but slightly soluble in water, darkened in colour by alkalies, and rendered a little more soluble. It dissolves in alcohol, and by evaporation forms micaceous crystalline plates; it is very slightly soluble in ether or volatile oils; with concentrated nitric acid, it yields a very intense green colour, disappearing by solution, re-appearing by concentration; alkalies and all oxygenating bodies make it disappear entirely. Sulphuric acid also produces a green colour with this substance; muriatic acid has no action. It has no action on the animal economy."

The account of these investigations is published in the *Annales de Chimie*, xxvi.

---

### MISCELLANEOUS.

15. *On Pumping, as a Punishment for unmanageable Lunatics.*—In a paper by Dr. Bardsley, of Manchester, containing some account of the Salpetriere, the following remarks occur upon this subject:—

When every other attempt to enforce tranquillity and mild behaviour on the part of some of the more mischievous and unmanageable patients, has failed, pumping is occasionally employed as a last resource, and sometimes with the desired success. This mode of punishment is somewhat harsh; but, if applied with due care and moderation, it will be found not altogether undeserving of trial. In the following case, as in several others, its use was attended with a favourable result.

A young brunette, of robust habit of body, had been for several years subject to violent exacerbations of insanity, for a week or ten days preceding the menstrual discharge. Threats, bodily punishment, and various modes of coercion, had been employed to deter her from unremitting attempts to commit suicide: but on several occasions she had nearly succeeded in her dreadful purpose. At the desire of her friends, she was admitted into the Salpetriere. As soon as this state of fury supervened, she was ordered to be conducted to the bath, and to have cold water applied to the head in the manner above alluded to. Its effects were striking; for, though a moment before she was vehemently vociferating execrations against her friends and all present, and using violent means to extricate herself from her novel situation, when the shock came she seemed in a moment to have lost both mental and physical force. In about ten minutes the paroxysm returned, and the affusion was again repeated, with a similar result. It was found unnecessary, the succeeding month, to resort to this practice; for, during the height of her excitement, the mere mention of the bath was sufficient to check it, and render her tranquil and composed.—*(Edinburgh Medical and Surgical Journal.)*

16. *Preparation of Caoutchouc.*—Mr. T. HANCOCK has succeeded, by some process, (the results of a long investigation, but which he has not published,) in working caoutchouc with great facility and readiness. It is cast, as we understand, into large ingots or cakes, and being cut with a wet knife into leaves or sheets, about an eighth or a tenth of an inch in thickness, can then be applied to almost any purpose for which the properties of the material render it fit. The caoutchouc thus prepared is more flexible and adhesive than that which is generally found in the shops, and is worked with singular facility. Recent sections made with a sharp knife or scissors, when brought together and pressed, adhere so firmly as to resist rupture as strongly as any other part, so that, if two sheets be laid together and cut round, the mere act of cutting joins the edges, and a little pressure on them makes a perfect bag, of one piece of substance. The adhesion of the substance in those parts where it is not required, is entirely prevented by rubbing them with a little flour, or other substance in fine powder. In this way flexible tube catheters, &c. are prepared; the tubes being intended for experiments on gases, and where occasion might require they should sustain considerable internal pressure, are made double, and have a piece of twine twisted spirally round between the two. This, therefore, is imbedded in the caoutchouc, and, at the same time that it allows of any extension in length of the tube, prevents its expanding laterally. The caoutchouc is, in this state, exceedingly elastic. Bags made of it, as before described, have been expanded, by having air forced into them, until the caoutchouc was quite transparent; and, when expanded by hydrogen, they were so light as to form balloons, with considerable ascending power; but the hydrogen gradually escaped, perhaps through the pores of this thin film of caoutchouc. On expanding the bags in this way, the junctions yielded like the other parts, and ultimately almost disappeared. When cut thin, or when extended, this substance forms excellent washers, or collars for stop-cocks, very little pressure being sufficient to render them perfectly tight. Leather has also been coated on one surface with the caoutchouc; and, without being at all adhesive, or having any particular odour, is perfectly water-tight. Before caoutchouc was thus worked, it was often observed how many uses it might in such a case be applied to: now that it is so worked, it is surprising how few the cases are in which persons are induced to use it. Even for bougies and catheters it does not come into use, although one would suppose that the material was eminently fitted for the construction of these instruments.—*(Quarterly Journal.)*

17. *Cure for the contagious Glanders.*—Mr. SEWEL, assistant professor of the Veterinary College, Camden Town, well known especially for his happy discovery of curing in many cases, and in all relieving, that hitherto irremediable disease, the *foundered feet* of horses, by excision of the nerve, has been equally fortunate in another hitherto incurable disease, the *contagious glanders*. For this formidable malady, he has found a remedy in the use of sulphate of copper, given in the form of bolus or ball, daily, for several weeks. The dose was one to two ounces of sulphate of copper.

The committee of the Veterinary College, duly sensible of the value of Mr. Sewel's discoveries, have already voted him their thanks, with a handsome augmentation of his salary, and a promise of further rewards. —(*Philosophical Magazine.*)

18. *Local Causes of Carious Teeth and Odontalgia.*—It has been lately remarked, that caries and pain of the teeth prevail locally, more or less in different districts: about East Grinstead, Tunbridge Wells, Hartfield, and all that part of Sussex, it is particularly common,—scarcely any person above thirty having many sound teeth; while in other places this malady is very rare. To what is this to be ascribed? —(*Ibid.*)

---

### MONTHLY CATALOGUE OF MEDICAL BOOKS.

### METEOROLOGICAL JOURNAL,

*From September 20, to October 19, 1824.*

By Messrs. William Harris and Co. 50, Holborn, London.

| | MOON. | Rain gauge. | THERM. | | | BAROM. | | DE LUC'S HYG. | | WIND. | | ATMOSPHERIC VARIATIONS. | | |
|---|---|---|---|---|---|---|---|---|---|---|---|---|---|---|
| | | | 9 A.M. | MAX. | MIN. | 9 A.M. | 10 P.M. | 9 A.M. | 10 P.M. | 9 A.M. | 10 P.M. | 9 A.M. | 2 P.M. | 10 P.M. |
| Sep. | | | | | | | | | | | | | | |
| 20 | | | 55 | 65 | 53 | 29.75 | 29.80 | 95 | 90 | SSW | N | Rain | Fair | Fine |
| 21 | | | 54 | 62 | 52 | 29.77 | 29.80 | 95 | 96 | NW | NW | | Rain | Rain |
| 22 | ● | | 61 | 65 | 55 | 30.00 | 30.00 | 80 | 87 | NE | NE | Fine | Fine | Fine |
| 23 | | .50 | 55 | 60 | 55 | 29.90 | 29.91 | 89 | 83 | NW | NW | Rain | | |
| 24 | | .21 | 56 | 63 | 50 | 29.97 | 29.97 | 80 | 86 | NE | N | Fair | Rain | |
| 25 | | | 52 | 62 | 41 | 29.96 | 29.85 | 84 | 82 | N | W | Cloud. | | |
| 26 | | | 47 | 51 | 41 | 29.95 | 29.80 | 80 | 84 | NW | NW | Fine | Fine | |
| 27 | | .14 | 46 | 53 | 37 | 29.60 | 29.50 | 87 | 80 | WNW | NW | Rain | | |
| 28 | | | 43 | 50 | 37 | 29.73 | 29.85 | 78 | 79 | W | W | Fine | | Foggy |
| 29 | ☽ | | 48 | 57 | 49 | 29.84 | 29.77 | 73 | 79 | SE | S | Foggy | | Fine |
| 30 | | | 57 | 66 | 54 | 29.57 | 29.41 | 92 | 80 | S | SE | Cloud. | Fine | Rain |
| Oct. | | | | | | | | | | | | | | |
| 1 | | | 58 | 64 | 55 | 29.16 | 29.21 | 88 | 80 | SE | SW | Rain | Rain | Fine |
| 2 | | .50 | 58 | 67 | 51 | 29.30 | 29.42 | 75 | 80 | SW | SW | Cloud. | Fine | Fine |
| 3 | | | 57 | 60 | 50 | 29.70 | 29.77 | 80 | 82 | SW | S | Fine | Cloud. | |
| 4 | | | 56 | 62 | 46 | 29.74 | 29.74 | 88 | 84 | E | ESE | Fine | | |
| 5 | | | 55 | 60 | 57 | 29.57 | 29.48 | 90 | 96 | SE | E | Rain | Sho'ry | Sleet |
| 6 | | | 58 | 62 | 58 | 29.36 | 29.27 | 97 | 96 | SE | E | Cloud. | Rain | Cloud. |
| 7 | | .40 | 60 | 62 | 57 | 29.20 | 29.23 | 96 | 88 | SW | SSW | Cloud. | Sho'ry | |
| 8 | ○ | | 58 | 63 | 54 | 29.27 | 29.32 | 86 | 76 | SW | WSW | Fine | Fine | |
| 9 | | | 57 | 62 | 56 | 29.46 | 29.54 | 82 | 70 | W | W | Fine | | |
| 10 | | | 58 | 50 | 51 | 29.50 | 29.13 | 80 | 96 | E | S | Cloud. | Rain | |
| 11 | | .68 | 54 | 57 | 47 | 28.95 | 28.90 | 92 | 75 | SE | SW | Rain | | |
| 12 | | | 51 | 54 | 37 | 28.82 | 28.97 | 90 | 87 | NE | N var. | Cloud. | | |
| 13 | | | 43 | 46 | 35 | 29.30 | 29.42 | 75 | 66 | NW | WSW | Fine | | |
| 14 | | | 38 | 44 | 33 | 29.48 | 29.55 | 70 | 72 | WSW | W | Foggy | Fine | Sl.Fog |
| 15 | ☾ | | 42 | 47 | 32 | 29.57 | 29.62 | 77 | 82 | W | N | Foggy | | Cloud. |
| 16 | | | 37 | 45 | 35 | 29.78 | 29.87 | 78 | 64 | WNW | NW | Fine | | |
| 17 | | | 35 | 39 | 32 | 29.93 | 30.03 | 80 | 78 | W | SSE | Fine | | Cloud. |
| 18 | | .30 | 37 | 50 | 41 | 30.03 | 30.03 | 78 | 78 | WSW | WSW | Fine | | |
| 19 | | | 49 | 55 | 51 | 30.03 | 29.95 | 82 | 75 | WSW | WSW | Fine | | |

The quantity of rain fallen in the month of September, was 3 inches and 77.100ths.

